# Marital history, health and mortality among older men and women in England and Wales

**DOI:** 10.1186/1471-2458-10-554

**Published:** 2010-09-15

**Authors:** Emily MD Grundy, Cecilia Tomassini

**Affiliations:** 1 Centre for Population Studies, London School of Hygiene & Tropical Medicine, London, UK

## Abstract

**Background:**

Health benefits of marriage have long been recognised and extensively studied but previous research has yielded inconsistent results for older people, particularly older women. At older ages accumulated benefits or disadvantages of past marital experience, as well as current marital status, may be relevant, but fewer studies have considered effects of marital history. Possible effects of parity, and the extent to which these may contribute to marital status differentials in health, have also been rarely considered.

**Methods:**

We use data from the Office for National Statistics Longitudinal Study, a large record linkage study of 1% of the population of England & Wales, to analyse associations between marital history 1971-1991 and subsequent self-reported limiting long-term illness and mortality in a cohort of some 75,000 men and women aged 60-79 in 1991. We investigate whether prior marital status and time in current marital status influenced risks of mortality or long term illness using Poisson regression to analyse mortality differentials 1991-2001 and logistic regression to analyse differences in proportions reporting limiting long-term illness in 1991 and 2001. Co-variates included indicators of socio-economic status at two or three points of the adult life course and, for women, number of children borne (parity).

**Results:**

Relative to men in long-term first marriages, never-married men, widowers with varying durations of widowerhood, men divorced for between 10 and twenty years, and men in long-term remarriages had raised mortality 1991-2001. Men in long-term remarriages and those divorced or widowed since 1971 had higher odds of long-term illness in 1991; in 2001 the long-term remarried were the only group with significantly raised odds of long-term illness. Among women, the long-term remarried also had higher odds of reporting long-term illness in 1991 and in 2001 and those remarried and previously divorced had raised odds of long-term illness and raised mortality 1991-2001; this latter effect was not significant in models including parity. All widows had raised mortality 1991-2001 but associations between widowhood of varying durations and long-term illness in 1991 or 2001 were not significant once socio-economic status was controlled. Some groups of divorced women had higher mortality risks 1991-2001 and raised odds of long-term illness in 1991. Results for never-married women showed a divergence between associations with mortality and with long-term illness. In models controlling for socio-economic status, mortality risk was raised but the association with 1991 long-term illness was not significant and in 2001 never-married women had *lower *odds of reporting long-term illness than women in long-term first marriages. Formally taking account of selective survival in the 20 years prior to entry to the study population had minor effects on results.

**Conclusions:**

Results were consistent with previous studies in showing that the relationship between marital experience and later life health and mortality is considerably modified by socio-economic factors, and additionally showed that taking women's parity into account further moderated associations. Considering marital history rather than simply current marital status provided some insights into differentials between, for example, remarried people according to prior marital status and time remarried, but these groups were relatively small and there were some disadvantages of the approach in terms of loss of statistical power. Consideration of past histories is likely to be more important for later born cohorts whose partnership experiences have been less stable and more heterogeneous.

## Background

Numerous studies have found that married people have better health and lower mortality than the unmarried, with many showing the worst health and mortality among the formerly married [1-6]. These associations are widely attributed to a mixture of health protective, health selective and crisis induced effects. Protective effects of marriage include availability of social, emotional and instrumental support; better regulation of health related behaviours, which may be particularly important for men; and economic benefits, such as economies of scale and access to the partner's resources, which may be particularly important for women in older cohorts [7-10]. Selection effects-factors influencing both marital status and health and mortality- are potentially important as poor health, and characteristics and behaviours related to health, may reduce chances of marriage (and remarriage) and increase risks of both divorce and widowhood, in the latter case partly because of homogamy in the health status of couples [11-16]. Widowhood and divorce involve not just an end to benefits associated with marriage, but also the stress of the event itself which some studies have shown to be associated with adverse changes in health related behaviours, health, or mortality risks [12,17-19]. Most studies of marital status and health or mortality in older age groups have considered only current marital status and so ignore life course and accumulated benefits and risks of marital status trajectories. The aim of this paper was to investigate whether prior marital status, and time in current marital status, were associated with the subsequent health and mortality of men and women included in a large nationally representative record linkage study.

### Marital status, health and mortality at older ages

Challenges and transitions associated with ageing, such as increased risks of health limitations, reductions in income, retirement from paid work and, for parents, the departure of children from the home, would suggest that benefits from the support of a spouse - and so health differences between married and non-married people- might be potentially greater for older than for younger people. For the widowed and divorced the crisis effects of bereavement or marital breakdown may present additional challenges detrimental to health. Widowhood is a common event at older but not younger ages, particularly for women, this means that people widowed at older ages are a much less selected group than those widowed earlier in life. The potential availability of a larger peer group who have also experienced bereavement may also mean that older widows and widowers are able to draw on support from age peers to a greater extent than those widowed at younger ages. These two factors might suggest that the implications of widowhood for health should be less marked at older than at younger ages but reverse considerations may apply to older divorced people (as divorce is less usual at older than younger ages, particularly in the cohorts we consider here). Countervailing influences include greater risks of vulnerability in other domains of life, and perhaps greater difficulties in adapting to widowhood or divorce for those who have spent most of their adult life in a couple, both factors which would suggest that the stress of marital dissolution might be particularly severe in older age groups [20].

Given these considerations, it is surprising that empirical evidence of associations between marital status and indicators of health appears much weaker for older than for younger age groups, particularly for women and particularly in studies of health rather than mortality. Several studies of older British or US populations, for example, have found that at older ages never-married women have as good or better health than their married counterparts [5,21-23] or have found no health advantages for older women living with a spouse compared with those living alone [24,25]. Other studies have reported lower mortality among widows compared with married women [26,27] or found differences between married and some groups of unmarried men, but not others [12,28,29]. Nevertheless, a recent systematic review and meta analysis of studies of marital status and mortality in elderly age groups conducted since 1994 reported that mortality risks for the widowed and never-married were both about 10% higher than for the married, and that risks for the divorced and separated were slightly higher still, although not significantly different from those of the other unmarried groups [30]. In contrast to results from studies of younger groups which consistently suggest that the health benefits of marriage are greater for men than women, this analysis found no gender difference in the strength of associations. However, a subsequent very large study of 8 European countries, based on national population sources, found larger differentials among men than women and also that absolute (but not relative) differentials in mortality by marital status increased with age, being greatest in the older age groups among which most deaths occur [6]. This study also found that differentials varied by type of data source. In the countries for which population register data were available, observed excess mortality of the divorced was considerably less than in populations for which data were drawn from unlinked census and vital registration sources, a difference attributed to probable numerator denominator errors in reporting of marital status in the census and at registration of death [6].

Other methodological and measurement problems may also contribute to inconsistencies in results reported from previous research. In survey based studies, bias arising from non-response rates, sometimes in excess of 30%, and high levels of attrition in some longitudinal studies is a factor, especially as survey response is associated with both marital status and health related behaviour [31]. Lack of statistical power, particularly to detect differences between unmarried groups, is an acknowledged problem in many studies [12,21,28]. Exclusion of the institutional population is a further potential bias in many studies, including the meta analysis referred to above, [31] as both marital status and health are associated with institutional admission [5]. Differences in outcome measures used and the extent of control for initial health status, risk related behaviour, socio-economic resources and indicators of social support, as well as differences between populations, cohorts and age groups studied, may also account for some of the variation in results in previous research on the effects of marriage. Additionally, despite growing recognition of the importance of life course perspectives on later life and increasing interest in the effects of marital trajectories [32], most studies of older age groups have only considered associations between indicators of health and current marital status, or marital status transitions observed over relatively short time periods. As recognised in some more recent studies, past marital experience may also have implications for health and mortality, particularly at older ages. For example, accumulated benefits from healthier behaviours and lifestyles associated with marriage might be expected to have continuing effects, even after widowhood or divorce, and in some cases the formerly married may also able to draw on beneficial legacies of marriage, such as acquired marital assets and social support from children [10].

It might be expected that these beneficial legacies of past marriage would be evident in later life and there is some evidence to support this hypothesis from studies which have examined duration effects [17,33,34]. One British study, for example, found that the health of adults in early old age was positively related to the proportion of life spent married [35] and other studies of US or UK populations suggest chronic or cumulative effects of widowhood or divorce [21,23,36].

A further limitation of much previous research on marital status and health and mortality is that few studies have taken account of fertility histories, even though a growing body of research points to associations between this aspect of the life course and later life health and mortality [37-40].

In this study we use data from a large record linkage study of England and Wales to analyse associations between current and past marital status and later life mortality and health in a cohort of men and women aged 60-79 in 1991. The key research questions we address are whether prior marital status and time in current marital status influenced risks of mortality 1991-2001 and long-term illness in 1991 and 2001 and how far effects were modified or amplified by consideration of socio-economic characteristics and, for women, by fertility history.

## Methods

We use data from the Office for National Statistics Longitudinal Study (ONS LS), a record linkage study of approximately 1% of the population initially based on those enumerated in the 1971 Census of England and Wales and now including linked information from subsequent censuses in 1981, 1991 and 2001. Information on vital events, including deaths of sample members and of their spouses, has also been linked to the study. Strengths of the data set include large sample size, low non-response and attrition bias (as census coverage is good and rates of linkage high); inclusion of the institutional population and information spanning several decades of sample members' lives. Full details of the study have been reported elsewhere [41,42].

In this analysis we use data on some 33,700 male and 41,340 female LS members aged 60-79 in 1991 who had been present in the sample since 1971 (when aged 40-59). We derived a classification of marital history 1971-91 which we used in analysis of differentials in reported limiting long-term illness in 1991 and in mortality 1991-2001. Additionally we analysed differentials in long-term illness in 2001 in surviving members of the same cohort using an indicator of marital history over the previous thirty years (1971-2001). Exclusions from the 1991 sample included the very small proportion (less than 0.5%) in institutions in 1971; those absent from their usual residence in 1971 or 1981 (6%) for whom information on relevant co-variates was lacking, and 1% with missing or inconsistent marital status variables 1971-1991. Some 900 (1.2%) sample members were recorded as emigrants during the 1991-2001 follow-up period (and were excluded from the mortality analysis at point of exit) and 25,550 (34.0%) died leaving an eligible sample of 48,577 in 2001. Of these 774 (1.6%) were not found in the 2001 Census (as a result of undocumented emigration, linkage failure or other loss to follow-up); 516 (1.1%) had missing or inconsistent 2001 marital status information and 2,749 (5.7%) were excluded because of missing information on long-term illness in 2001, leaving an analysis sample of 44,538.

### Marital status/history

We used information collected in the 1971, 1981 and 1991 Censuses, together with information available on widowhoods to derive a classification of marital status and history 1971-91 and, for survivors, 1971-2001. Each census included a question on current de jure marital status, albeit in varying degrees of detail. The 1971 marital status question, for example, did not distinguish between first and subsequent marriages (although married women aged 16-59 were asked if they had been previously married in another section of the census). Subsequent censuses have asked whether people were in a first or a subsequent marriage but not required remarried people to specify marriage order. However, as the ONS LS includes information on all household members at each census we were able to use information on spouses' details to check whether those recorded as remarried in consecutive censuses were in all probability married to the same spouse. Men recorded as married (order unspecified) in 1971 and in their first marriage in 1981 were assumed to also have been in their first marriage in 1971. Prior to 2001, separated people were instructed to record themselves as married but information collected elsewhere in the census (particularly questions on relationships in the household) means that is possible to identify separated people in earlier censuses who here have been grouped with the divorced. Non- married people living with a partner (8% of non-married men and 4% of non-married women in 1991) were classified according to reported marital status rather than living arrangement as widowhood records can only be linked for the legally married.

Thus, although vital registrations of marriages and divorces are not linked to the ONS LS, the information available is sufficient to identify changes in marital status over a decade, although not to precisely time events. For example, we inferred that people in a first marriage in 1981 and remarried in 1991 with no intervening widowhood record must have experienced a divorce. Some recoding was undertaken in response to inconsistencies in reported marital statuses. Thus a small proportion of people who reported being married in one census and never-married in a subsequent one were classified as having experienced divorce (provided there was no intervening widowhood) as it is known that some divorced people revert to reporting themselves as never-married [43]. Similarly, people who reported being in a first marriage in one census but remarried in a previous census were recoded to remarried.

### Marital history classification

We distinguished 12 categories of marital status/history comprising: those in first marriages of at least 20 years duration in 1991 (or 30 years duration in 2001); those in first marriages contracted since 1971; those in second or subsequent marriages of at least 20 years duration (30 years in 2001); those in remarriages of shorter durations distinguishing people who had been widows or widowers prior to the remarriage from divorcés or divorcées; current widows and widowers with varying durations of widowhood (> 20 years, 10-19 years, < 10 years in 1991; > 30 years, 10-29 years or < 10 years in 2001); currently divorced people with equivalent varying durations of divorce, and those who were never-married. In the 2001 analysis all those divorced since 1971 were grouped together because of small numbers who had divorced in the previous 10 years.

### Outcome measures

The outcome measures we examine are all cause mortality 1991-2001 and self-reported long-term illness limiting activities in 1991 and 2001. Information on deaths comes from linked registration information included in the database which has been estimated to be nearly 100% complete [41,42]. Information on limiting long-term illness was drawn from a question included in the 1991 and 2001 (but unfortunately not in earlier) Censuses which asked whether or not people had 'any long-term illness, health problem or handicap [1991]/disability [2001] which limits his/her daily activities or the work he/she can do'. Completion notes instructed that problems due to old age should be included.

### Co-variates used in the analysis

#### Socio-economic status

The socio-economic indicators we use are based on information on educational qualifications, occupational social class (men only), housing tenure, and household access to a car, all of which have been used extensively in British research on health differentials [44]. The educational status indicator was drawn from a 1971 Census question on qualifications equivalent to or higher than A' level (exams taken around age 18); this unfortunately only distinguishes the most qualified ten to twenty percent of the population in the age groups we consider here. For men, we derived a social class score using information on current or last job recorded in the 1971 and 1981 Censuses. A score of 0 at each time point was assigned to those with no current or previous occupation; 1 point was allocated for an unskilled or partly skilled job (Registrar General's Social Classes V and IV); 2 for a skilled job (Social Classes IIIN and IIIM); and 3 for professional and managerial positions (Social Classes I and II). This gave a total score ranging from 0 (lowest- no occupation at either time point) to 6 (highest- professional/managerial at both time points). In the analysis those with a score of 0 and those with a score of 1 (not working at one time point and in an unskilled or partly skilled job at the other) were combined as both these categories were small. 1991 data were not used in this derivation as by then most of the men we consider were not working. As only a third of women in the sample were in employment in both 1971 and 1981, it was not sensible to derive this measure for women.

We used a similar approach to derive a tenure/car score allocating 2 points for home ownership and 1 point for household possession of a car at the 1971, 1981 and 1991 Censuses. This gave a total score ranging from 0 (lowest- not a home owner and no car at any census) to 9 (home and car owner at all three time points). The small numbers in an institution in 1981 or 1991 were classed as non car and home owners. In analyses of outcomes in 2001 we also included an indicator of home ownership in 2001. We did not include 2001 car ownership as access to a car at ages beyond 75 may be more strongly associated with health and, for women, with marital status, than with socio-economic status.

#### Parity

In additional analysis for women, we investigated effects of parity using retrospective fertility data collected in the 1971 Census from women who were then ever-married and aged 16-59, together with small numbers of subsequent births linked to the data set. This measure does not include non-marital births prior to 1971 and also assumes that women who were never-married in 1971 were then childless, but non-marital childbearing in the early and mid decades of the 20^th ^century was relatively unusual in England and Wales, accounting for only 4-6% of all births per year. Details of this derivation and assessment of the quality of the data have been reported elsewhere [39].

### Analysis

We wanted to see how associations between marital status/history were modified by consideration of socio-economic status (prior to outcome) and, in the case of women, parity. We therefore fitted models including marital status/history only; marital status/history and socio-economic characteristics and, for women, models including parity as well as the socio-economic indicators. In order to assess what additional insights were gained from considering marital history, we also present results from models including current marital status only. All models included age in single years entered as a continuous variable. The tenure/car and social class scores were linearly associated with outcome variables and were also entered as continuous variables; all other variables were categorical. We used Poisson regression to analyse mortality (deaths/person years of exposure) during the ten year follow-up period. Poisson regression was chosen as the most appropriate method of analysis for the mortality data because of the size of the data set and the ability this method provides to analyse data files in collapsed (aggregated) form. This enabled us to comply with stringent confidentiality requirements, under which analyses including counts of one or two are not allowed to leave the ONS safe setting, while retaining the level of detail desired. A further advantage is that Poisson regression models can be fitted to data sets including tied failure times, common in data sets of this size, whereas in Cox regression models this is problematic. Results from the two kinds of models in analyses, such as those presented here, in which follow-up times are split into small intervals (in this case fractions of person years of exposure equivalent to days of follow-up) are effectively the same [45-47]. Logistic regression was used to analyse the binary health outcomes.

## Results

Table 1 shows the distribution of the samples at 1991 and 2001 by marital history and other variables used in the analysis. Mean ages of the samples of men and women in 1991 were 68 and 69 respectively. Over two thirds of the men and just under a half of the women were in their first marriage and had been married for at least twenty years. Some ten percent of men and seven percent of women were in a subsequent marriage. As would be expected, a much larger proportion of women than men were widowed, and most had been widowed for less than ten years. Well known gender differences in educational opportunities (in these cohorts) and acquisition of resources are reflected in differences in the proportions with educational qualifications and in mean tenure/car scores. Variations in the characteristics of the sample at each time point reflect ageing, period differences, and differential survival. By 2001, when surviving sample members were aged 70-89, just over half of the women were widows although among men those in first marriage still predominated. The proportion reporting long-term illness was higher in 2001 than in 1991. This reflects not just ageing of the sample, but also the fact that the reported prevalence of long-term illness was higher for all age groups in 2001 than in 1991. The mean age of survivors in 2001 (76 for men and 78 for women) was slightly less than ten years older than mean age in 1991 and tenure/car and social class scores, and proportions with higher level qualifications, were slightly higher in the 2001 survivor sample than among those included in 1991.

**Table 1:** Distribution of men and women aged 60-79 in 1991 by marital, socio-demographic and health characteristics used in the analysis, 1991 and 2001.

		*1991*		*2001*
	***Men***	***Women***	***Men***	***Women***

Age: mean (SD)	67.98 (5.43)	68.76 (5.58)	76.42 (4.85)	77.52 (5.21)
*Marital status/history 1971-91/1971-2001*				
1st marriage -long term (pre 1971)	68.68	47.87	60.68	31.46
1^st ^marriage since 1971	0.86	0.42	0.98	0.30
*All in 1^st ^marriage*	*69.54*	*48.29*	*61.66*	*32.40*
Remarried - long term (pre 1971)	5.53	2.44	3.95	1.26
Remarried since 1971, previously widowed	2.18	1.47	2.72	1.59
Remarried since 1971, previously divorced	2.48	2.57	2.20	1.46
*All remarried*	*10.19*	*6.48*	*8.87*	*3.60*
Widowed- long term (pre 1971)	0.79	5.16	0.46	4.03
Widowed- intermediate (1971-81/1971-91^a^)	2.20	10.21	6.61	26.04
Widowed- recent (post 1981/91^b^)	7.69	19.41	13.16	23.89
*All widowed*	*10.68*	*34.78*	*20.23*	*54.29*
Divorced- long term (pre 1971)	0.57	1.25	0.43	1.03
Divorced- intermediate (1971-81/post 1971^c^)	1.74	2.08	3.41	3.07
Divorced- recent (post 1981)	1.00	0.79		
*All divorced*	*3.41*	*4.12*	*3.84*	*3.75*
Never-married	6.17	6.34	5.40	5.87
*Socio-economic indicators*				
Educational qualification (1971)	13.73	8.25	16.07	9.22
Tenure/car score 1971-91, mean (SD)	5.85 (3.22)	5.31 (3.32)	6.32 (3.04)	5.68 (3.26)
Social class score 1971-81, mean (SD)	3.94 (1.45)	--	4.11 (1.40)	--
Owner occupier (2001)			73.57	65.48
Has long-term illness	34.97	31.85	52.09	55.97

N	33,686	41,341	17,997	26,541

^a^1971-81 in analysis of 1991-2001 mortality and 1991 long-term illness; 1971-91 in 2001 analysis.

^b ^1981-91 in analysis of 1991-2001 mortality and 1991 long-term illness; 1991-2001 in 2001 analysis.

^c ^1971-81 in analysis of 1991-2001 mortality and 1991 long-term illness; post 1971 in 2001 analysis

**Figure 1:**
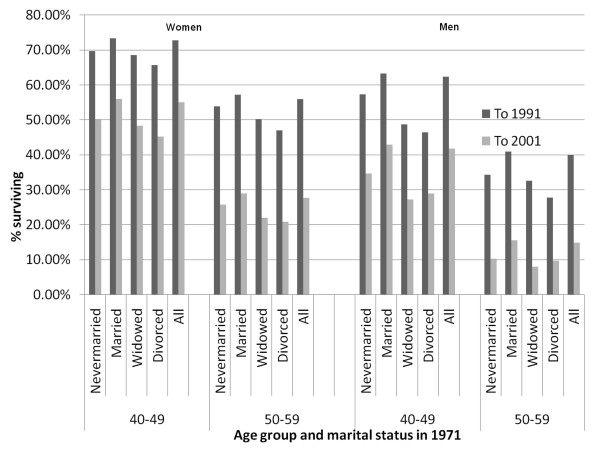
**Proportions of the 1971 study population alive and in the sample in 1991 and 2001 by gender, age group and marital status in 1971**.

Sample characteristics in 1991 (and 2001) will also reflect differentials in mortality prior to 1991, including variations by marital status. This is illustrated in Figure 1 which shows what proportion of 1971 study members survived to be included in the analyses reported here by gender, age group and marital status in 1971. For men and women in both age groups considered, the proportions of survivors to 1991 and 2001 were highest for those who were married in 1971 and lowest for those then widowed or divorced. Thus 46% of divorced men aged 40-49 in 1971 survived to be included in our 1991 analysis and only 29% to 2001; equivalent proportions among married men of the same age were 63% and 43%. Possible implications of this prior selection of the samples analysed here are considered further in the Discussion section of this paper.

### Marital history and mortality 1991-2001

Results of the analysis of mortality 1991-2001 are presented in Table 2. Results from two models are shown. Model 1 included age and the marital history variable; Model 2 additionally included the socio-economic indicators. Results from equivalent models including current marital status, rather than marital history, are also shown at the bottom of the table. Compared with those in long-term first marriages, men in all non-married categories and those remarried by 1971, but not those who had married or remarried since 1971, had raised mortality. Excess risks for all non-married groups were reduced when education, social class score and tenure/car score were included and ceased to be statistically significant for the small numbers of long-term divorced or those divorced after 1981 (Model 2). The highest relative mortality was among widowers who had been widowed for more than ten years (with no differences between those widowed for 10-19 years and those widowed for longer) and for those divorced for between 10-19 years. Women divorced within the last ten years had the highest mortality relative to women in long-term first marriages. Mortality was also raised among other divorced women, all widows, the never-married and the currently remarried who had previously been divorced, but not among those who had remarried following widowhood or those already remarried by 1971 (who prior marital status cannot be determined). When socio-economic status variables were included in the model, the size of these effects was reduced and ceased to be significant for women divorced between 10 and 19 years. Results by current marital status showed raised mortality for all groups of non-married men and women and also for the remarried, although in the case of men mortality risk was only raised significantly when socio-economic factors were controlled (Model 2). For groups other than remarried men, adjustment for socioeconomic factors tended to slightly reduce excess risks, as in the analysis using the marital history categories.

**Table 2:** Rate ratios (95% confidence intervals) from Poisson regression analysis of male and female mortality 1991-2001.

	*Men*				*Women*			
	**Model 1**		**Model 2**		**Model 1**		**Model 2**	
	**IRR**		**IRR**		**IRR**		**IRR**	

Age	1.10***	(1.10-1.10)	1.10***	(1.09-1.10)	1.10***	(1.10-1.11)	1.10***	(1.10-1.11)
*Marital history* 1st marriage - long term (20+ years)	1.00		1.00		1.00		1.00	
1^st ^marriage- since 1971	0.83	(0.66-1.04)	0.81	(0.64-1.01)	1.24	(0.92-1.67)	1.23	(0.91-1.67)
Remarried - long term (20+years)	1.11**	(1.03-1.19)	1.13**	(1.05-1.21)	1.09	(0.97-1.23)	1.08	(0.96-1.21)
Remarried since 1971, previously widowed	0.92	(0.82-1.03)	0.93	(0.83-1.05)	1.03	(0.88-1.21)	1.00	(0.86-1.17)
Remarried since 1971, previously divorced	1.05	(0.93-1.18)	1.03	(0.92-1.15)	1.21**	(1.08-1.37)	1.16*	(1.03-1.31)
Widowed- long term (20+ years)	1.49***	(1.28-1.74)	1.34***	(1.15-1.56)	1.21***	(1.13-1.30)	1.10*	(1.02-1.18)
Widowed- intermediate (10-19 years)	1.46***	(1.33-1.61)	1.36***	(1.24-1.50)	1.19***	(1.12-1.26)	1.09**	(1.03-1.15)
Widowed- recent (< 10 years)	1.28***	(1.21-1.36)	1.20***	(1.14-1.27)	1.19***	(1.14-1.25)	1.12**	(1.07-1.17)
Divorced- long term (20+ years)	1.24*	(1.01-1.53)	1.11	(0.90-1.36)	1.38***	(1.19-1.61)	1.23**	(1.05-1.43)
Divorced- intermediate (10-19 years)	1.43***	(1.26-1.62)	1.30***	(1.15-1.48)	1.26***	(1.10-1.43)	1.13	(0.99-1.29)
Divorced- recent (< 10 years)	1.23*	(1.04-1.46)	1.15	(0.97-1.36)	1.61***	(1.32-1.95)	1.47***	(1.21-1.79)
Never-married	1.38***	(1.29-1.48)	1.22***	(1.14-1.31)	1.18***	(1.10-1.27)	1.12**	(1.04-1.20)
*Socioeconomic variables*								
Educational qual. 1971 (ref. none)			0.91***	(0.86-0.97)			0.84***	(0.78-0.91)
Tenure/car score 1971-91 (0-9)			0.95***	(0.95-0.96)			0.95***	(0.95-0.96)
Social class score 1971-81 (0-6)			0.96***	(0.95-0.97)				
*Current marital status*								
*All in 1^st ^marriage*	1.00		1.00		1.00		1.00	
*All remarried*	1.05	(0.99-1.11)	1.06*	(1.00-1.12)	1.12**	(1.04-1.21)	1.09*	(1.01-1.18)
*All widowed*	1.34***	(1.27-1.41)	1.25***	(1.19-1.31)	1.19***	(1.14-1.24)	1.11***	(1.06-1.15)
*All divorced*	1.34***	(1.22-1.47)	1.22***	(1.11-1.34)	1.36***	(1.24-1.49)	1.22***	(1.11-1.34)
*Never-married*	1.38***	(1.29-1.48)	1.22***	(1.14-1.31)	1.19***	(1.10-1.27)	1.12**	(1.04-1.20)

Number of deaths	13,296				12,254			

Notes: *p < 0.05; **p < 0.01; ***p < 0.001

For both men and women, associations between age and mortality were highly consistent in both models. Lacking a higher level qualification, each unit decrease in tenure/car score and each additional year of age all increased mortality risks. For men each unit change in the social class score was associated with a 4% change in the mortality risk ratio.

### Marital history and long-term illness in 1991 and 2001

Table 3 shows results from logistic regression models of presence of limiting long-term illness in 1991. As in the mortality analysis, all non-married men and men in long-term remarriages had higher odds of illness than those in long-term first marriage. However, when socio-economic status was controlled, odds for never-married men and the long-term divorced or widowed were no longer significantly raised, relative to men in long-term first marriages, and those for other categories of formerly married men were reduced, although still significant. Results from this model also showed that men who had married for the first time since 1971 had lower odds of long-term illness than those in long-term first marriages. For women, as for men, age adjusted results showed higher odds of illness for all non-married groups, those remarried by 1971 and, additionally, those remarried since 1971 following divorce. However, when socio-economic status was controlled, odds were significantly raised only for the recently divorced, the long-term remarried and those remarried since 1971 having previously been divorced.

**Table 3:** Odd-ratios (95% confidence intervals) from logistic regression analysis of long-term illness 1991 (ages 60-79)

	*Men*				*Women*			
	**Model 1**		**Model 2**		**Model 1**		**Model 2**	
	**OR**		**OR**		**OR**		**OR**	

Age	1.03***	(1.03-1.04)	1.03***	(1.02-1.03)	1.07***	(1.07-1.08)	1.07***	(1.06-1.07)
*Marital history*								
1st marriage - long term (20+ years)		1.00		1.00		1.00		1.00
1^st ^marriage- since 1971	0.77	(0.59-1.01)	0.74*	(0.56-0.96)	0.85	(0.60-1.22)	0.83	(0.58-1.19)
Remarried - long term (20+years)	1.24***	(1.12-1.36)	1.26***	(1.15-1.40)	1.40***	(1.22-1.60)	1.34***	(1.20-1.57)
Remarried since 1971, previously widowed	0.91	(0.77-1.06)	0.93	(0.79-1.09)	1.05	(0.88-1.25)	1.00	(0.83-1.19)
Remarried since 1971, previously divorced	1.05	(0.91-1.22)	1.01	(0.87-1.17)	1.25**	(1.09-1.43)	1.16*	(1.02-1.33)
Widowed- long term (20+ years)	1.33*	(1.04-1.70)	1.10	(0.86-1.41)	1.18***	(1.07-1.30)	1.01	(0.92-1.11)
Widowed- intermediate (10-19 years)	1.35***	(1.16-1.56)	1.18*	(1.02-1.38)	1.11**	(1.03-1.19)	0.96	(0.89-1.04)
Widowed- recent (< 10 years)	1.26***	(1.16-1.37)	1.12*	(1.03-1.22)	1.07*	(1.01-1.13)	0.96	(0.91-1.02)
Divorced- long term (20+ years)	1.40*	(1.05-1.87)	1.14	(0.85-1.53)	1.42***	(1.18-1.71)	1.15	(0.96-1.39)
Divorced- intermediate (10-19 years)	1.59***	(1.35-1.88)	1.35***	(1.14-1.60)	1.37***	(1.18-1.58)	1.15	(0.99-1.33)
Divorced- recent (< 10 years)	1.53***	(1.24-1.89)	1.39**	(1.13-1.72)	1.72***	(1.36-2.16)	1.49**	(1.18-1.88)
Never-married	1.22***	(1.11-1.34)	0.97	(0.88-1.06)	1.17**	(1.07-1.27)	1.04	(0.96-1.14)
*Socioeconomic variables*								
Educational qual.1971 (ref. none)			0.84***	(0.77-0.90)			0.88***	(0.79-0.94)
Tenure/car score 1971-91			0.94***	(0.93-0.94)			0.92***	(0.91-0.92)
Social class score 1971-81			0.89***	(0.87-0.90)		--		--
*Current marital status*								
*All in 1^st ^marriage*	1.00		1.00		1.00		1.00	
*All remarried*	1.12**	(1.04-1.20)	1.13**	(1.04-1.22)	1.26***	(1.15-1.37)	1.20***	(1.10-1.31)
*All widowed*	1.29***	(1.20-1.38)	1.14**	(1.06-1.22)	1.10***	(1.05-1.15)	0.97	(0.92-1.02)
*All divorced*	1.54***	(1.37-1.74)	1.33***	(1.18-1.51)	1.45***	(1.30-1.61)	1.21***	(1.09-1.35)
*Never-married*	1.22***	(1.11-1.34)	0.97	(0.88-1.06)	1.17**	(1.07-1.27)	1.04	(0.96-1.14)

*N*	33,686				41,341			

*p < 0.05; **p < 0.01; ***p < 0.001

Table 4 shows differences in reported presence of limiting long term illness in 2001. As detailed earlier, the classification of marital history in 2001 is based on status and changes covering a 30, rather than 20, year period and differs slightly from that used in 1991. Among men, those in long-term remarriages had raised odds of long-term illness and this association was unchanged when socio-economic status was controlled. Men widowed or divorced since 1971 also had raised odds of long-term illness in the age adjusted model (Model 1), but these associations ceased to be significant when socio-economic status was controlled (Model 2). For women results from Model 1 are similar to those reported for long-term illness in 1991, and for mortality, in showing higher odds of poorer health for the long-term remarried, those remarried and previously divorced, all widowed groups and those divorced since 1971, although odds for the never-married were not raised. However, when socio-economic status was controlled (Model 2) only women in long-term remarriages and those who had remarried since 1971 and had previously been divorced had raised odds of long-term illness, relative to women in long-term first marriages, and the odds ratio for never-married women was *lower *than for the reference group of women in long-term first marriages.

**Table 4:** Odd-ratios (95% confidence intervals) from logistic regression analysis of long-term illness 2001 (ages 70-89)

	*Men*				*Women*			
	**Model 1**		**Model 2**		**Model 1**		**Model 2**	
	**OR**		**OR**		**OR**		**OR**	

Age	1.07***	(1.06-1.08)	1.06***	(1.06-1.07)	1.10***	(1.09-1.10)	1.09***	(1.09-1.10)
*Marital history 1971-01*								
1st marriage -long term (30+ years)	1.00		1.00		1.00		1.00	
1^st ^marriage- since 1971	1.08	(0.80-1.45)	1.00	(0.74-1.36)	0.66	(0.41-1.06)	0.68	(0.42-1.09)
Remarried - long term (30+ years)	1.18*	(1.01-1.37)	1.18*	(1.01-1.38)	1.41**	(1.12-1.77)	1.36**	(1.08-1.71)
Remarried since 1971, previously widowed	1.04	(0.86-1.24)	1.03	(0.86-1.24)	1.18	(0.96-1.45)	1.11	(0.90-1.36)
Remarried since 1971, previously divorced	0.98	(0.80-1.20)	0.95	(0.78-1.17)	1.45**	(1.17-1.79)	1.34**	(1.08-1.66)
Widowed- long term (30+ years)	1.28	(0.81-2.03)	1.04	(0.65-1.66)	1.29**	(1.12-1.49)	1.12	(0.97-1.30)
Widowed- intermediate (10-29 years)	1.15*	(1.01-1.30)	1.00	(0.88-1.13)	1.19***	(1.07-1.27)	1.04	(0.97-1.12)
Widowed- recent (< 10 years)	1.14*	(1.04-1.26)	1.03	(0.94-1.14)	1.14***	(1.07-1.23)	1.05	(0.98-1.13)
Divorced- long term (30+ years)	1.00	(0.63-1.58)	0.86	(0.54-1.37)	1.25	(0.96-1.61)	1.03	(0.79-1.34)
Divorced- intermediate or recent (< 30 years)	1.24*	(1.05-1.46)	1.08	(0.91-1.28)	1.31***	(1.13-1.53)	1.09	(0.94-1.28)
Never-married	1.07	(0.94-1.23)	0.89	(0.77-1.01)	0.96	(0.86-1.08)	0.88**	(0.78-0.99)
*Socioeconomic variables*								
Educational qual.1971 (ref. none)			0.83***	(0.76-0.90)			0.82***	(0.75-0.90)
Tenure/car score 1971-91			0.97***	(0.96-0.98)			0.98***	(0.97-0.99)
Social class score 1971-8			0.93***	(0.90-0.95)				
Owner occupier 2001 (ref.yes)			0.75***	(0.68-0.82)			0.60***	(0.56-0.65)
*Current marital status*								
*All in 1^st ^marriage*	1.00		1.00		1.00		1.00	
*All remarried*	1.08	(0.97-1.20)	1.08	(0.97-1.20)	1.37***	(1.19-1.57)	1.30***	(1.13-1.49)
*All widowed*	1.15***	(1.06-1.24)	1.02	(0.94-1.11)	1.17***	(1.11-1.25)	1.06	(0.99-1.12)
*All divorced*	1.21*	(1.04-1.42)	1.05	(0.90-1.23)	1.29***	(1.12-1.49)	1.08	(0.94-1.25)
*Never-married*	1.07	(0.94-1.23)	0.89	(0.77-1.01)	0.98	(0.87-1.10)	0.88*	(0.78-0.99)

N	17,997		17,997		25,029		25,029	

*p < 0.05; **p < 0.01; ***p < 0.001

#### Parity

In Table 5 we present results from models of differentials in female mortality 1991-2001, and long-term illness in 1991 and 2001 which include parity. Relative to mothers of two children, women who had had five or more births had raised risks of mortality and of long-term illness in 1991 and 2001; in 2001 odds of long-term illness were also raised for women with three or four births. At the other end of the distribution, nulliparous women had raised mortality risks and women who had had only one birth had raised odds of long-term illness in 1991. Comparing the results shown in Table 5 with those shown for women in Table 2 (Model 2), shows that including parity in the modelling of mortality had no or trivial effects on estimates for widowed or divorced women, but some effect on the mortality risk ratio for remarried women who had previously been divorced, which ceased to be significantly raised. Odds of long-term illness in 1991 were also no longer significantly raised for remarried women who had previously been divorced, although they were still raised for 2001 long-term illness. Mortality risk ratios for never-married women were not raised when parity was included in the model and the reduced odds of 2001 long-term illness shown in Table 4 (Model 2) was also no longer significant in the model including parity. In interpreting this difference between models including and excluding parity it must be remembered that we have assumed that all women who were never-married in 1971 were nulliparous.

**Table 5:** Odd ratios and rate ratios (95% confidence intervals) from Poisson and logistic regression analysis of mortality 1991-2001 and long-term illness in 1991 and 2001 for all women by marital history and parity.

	Mortality 1991-2001		Long-term illness 1991		Long-term illness 2001	
	IRR		OR		OR	
Age	1.10***	(1.10-1.11)	1.07***	(1.06-1.07)	1.09***	(1.09-1.10)
*Marital status/history 1971-91/01*						
Long term first marriage	1.00		1.00		1.00	
First marriage since 1971	1.14	(0.84-1.54)	0.84	(0.58-1.20)	0.75	(0.46-1.20)
Long term remarriage	1.08	(0.96-1.21)	1.29***	(1.13-1.47)	1.35*	(1.08-1.67)
Remarried since 1971, previously widowed	1.03	(0.84-1.27)	1.12	(0.88-1.42)	1.15	(0.90-1.46)
Remarried since 1971, previously divorced	1.10	(0.98-1.23)	1.09	(0.97-1.23)	1.38**	(1.08-1.76)
Long-term widow (20+ years)^1^	1.10*	(1.02-1.18)	1.01	(0.92-1.11)	1.09	(0.94-1.27)
Widow (10-19 years)^2^	1.08**	(1.02-1.15)	0.96	(0.89-1.04)	1.03	(0.96-1.11)
Widow (< 10 years)	1.12***	(1.06-1.17)	0.96	(0.91-1.02)	1.04	(0.97-1.12)
Long-term divorced (20+ years)^1^	1.22**	(1.05-1.42)	1.15	(0.95-1.39)	1.02	(0.78-1.32)
Divorced (10-19 years)^3^	1.13	(0.99-1.29)	1.14	(0.98-1.33)		
Divorced (< 10 years)	1.46***	(1.20-1.78)	1.48**	(1.17-1.86)	1.07	(0.91-1.26)
Never-married	1.03	(0.95-1.12)	1.05	(0.95-1.17)	0.95	(0.83-1.09)
*Socioeconomic variables*						
Educational qual. 1971 (ref. none)	0.84***	(0.78-0.90)	0.87**	(0.80-0.95)	0.83***	(0.75-0.90)
Tenure/car score 1971-91	0.95***	(0.95-0.96)	0.92***	(0.91-0.93)	0.96***	(0.94-0.98)
Owner occupier 2001					0.62***	(0.58-0.67)
*Parity*						
0	1.16***	(1.09-1.22)	1.05	(0.98-1.13)	0.93	(0.86-1.02)
1	1.09**	(1.03-1.14)	1.09**	(1.02-1.16)	1.02	(0.94-1.09)
2 (ref)	1.00		1.00		1.00	
3	1.01	(0.96-1.08)	1.06	(0.99-1.13)	1.10*	(1.01-1.19)
4	1.05	(0.97-1.13)	1.05	(0.96-1.15)	1.18**	(1.06-1.32)
5+	1.15**	(1.06-1.23)	1.25***	(1.14-1.37)	1.40***	(1.24-1.58)

Number of deaths/N	12,254		41341		25,029	

## Discussion

Many investigations into marital status differentials in health or mortality at older ages have considered only current status or transitions over a short period, here we investigated associations between an indicator of marital history spanning twenty or thirty years and mortality and long-term illness in older age groups.

Results showed that relative to men in long-term first marriages, men in all non-married groups and those in long-term remarriages had raised mortality 1991-2001, although for the small proportions divorced before 1971 or after 1981 (and not remarried) this excess was not significant once socio-economic status was controlled. Men in long-term remarriages and those divorced or widowed since 1971 also had higher odds of long-term illness in 1991; in 2001, however, the long-term remarried were the only group with significantly raised odds of long-term illness. Women in long-term remarriages also had higher odds of reporting long-term illness in 1991 and in 2001, relative to women in long-term first marriages, and remarried women who had previously been divorced had both raised odds of long-term illness and raised mortality 1991-2001, although this latter effect ceased to be significant when parity was also considered. After control for socio-economic status, some groups of divorced women had higher mortality risks 1991-2001 and raised odds of long-term illness in 1991; mortality was also raised for all widows. Relative to women in long-term first marriages, never-married women had raised mortality risks but the association with 1991 long-term illness was not significant and in 2001 never-married women had *lower *odds of reporting long-term illness than women in long-term first marriages. Neither the positive association with mortality nor the negative one with long-term illness in 2001 remained significant when parity was also taken into account but a limitation of this analysis is that we had no information on non-marital births prior to 1971 and so, taking into account the low levels of non-marital childbearing in the relevant period, made the assumption that all women who were never-married in 1971 were then childless.

It is interesting that for both never-married men and women, the results show a divergence between associations with mortality risks, which were raised, and odds of long-term illness which were either not raised or, in the case of women in 2001, significantly reduced relative to those in long-term first marriages. This is consistent with the literature in that several studies have found that older never-married women report similar or better health than married women although many studies have found higher mortality for never-married compared with married groups [5,21-23,30]. The measure of health status used in this study - self-reported illness limiting daily activities- is a subjective one and it is possible that people's perceptions of health problems or the limitations they produce may vary with marital status. It has been suggested, for example, that some of the health benefits of marriage result from monitoring of health by a spouse and also that those with fixed obligations that cannot be reassigned may be less likely to adopt the 'sick role' [1]. Possibly, never-married older people are less aware of health changes and may have a higher threshold for reporting illness. The subjective nature of the health measure used also makes it difficult to interpret the higher age specific prevalence rates of reported limiting long-term illness in 2001 compared with 1991 referred to earlier. Although it is possible that this indicates a real deterioration in population health, it may reflect increases in people's health expectations and consequent greater propensity to report health limitations.

In terms of what consideration of marital history, rather than just current marital status, adds to our understanding and knowledge, this is clearest for the remarried. Although those in the various categories of remarried considered accounted for only small proportions of the study samples, the remarried as a group outnumbered the never-married for both men and women in 1991 and for men in 2001 and will be larger in more recent cohorts, so understanding possible implications for health is important. We found that men in long-term remarriages contracted before 1971 had higher mortality and higher odds of reporting long-term illness than men in long-term first marriages whereas men remarried since 1971 generally had better health and lower mortality, although this only reached conventional levels of statistical significance in the analysis of 1991 long-term illness.

In the comparable analyses for women, the long-term remarried had higher odds of reporting long-term illness in 1991 and 2001, relative to women in long-term first marriages, and those remarried following divorce had significantly worse health and mortality in all analyses. No such disadvantage was evident for remarried women who had previously been widowed. Those already remarried by 1971 would have experienced marital dissolution at a relatively young age, possibly the disruption to life course trajectories resulting from this may have enduring health consequences. One such effect may be reduced opportunities for having children and in the case of women who had remarried following divorce our results showed that apparent health disadvantages tended to weaken or disappear when parity was controlled. It is also possible that relatively early marital termination, particularly through divorce, and remarriage in these cohorts is associated with risk taking and unhealthy lifestyles which unfortunately we had no information on. However, we did not find any equivalent consistent disadvantage among women divorced before 1971 who had not remarried, whose characteristics are likely to be even more unfavourable than women who remarried after divorce. This group was relatively small (517 women in 1991 and 342 in 2001) and the power of the analysis consequently weaker.

Among the much larger proportions of widows and widowers, there were no clear and consistent differences between those with the differing durations of widowhood we considered. Many studies of widowhood have suggested that excess mortality is most pronounced within the first year following bereavement, with less or no excess risk at longer durations of widowhood [48,49]. However, Manzoli and colleagues found little evidence for such an effect in their meta analyses of studies since 1994 and suggested this was mainly a feature of older studies [30]. We did not separately identify the very recently widowed because it was not possible to identify a similar group of very recently divorced people and small numbers would have further limited power of the analysis.

Controlling for socio-economic status, which was strongly associated with mortality and health, considerably modified associations found, particularly for women and particularly for the never-married. This indicates a greater co-variance of marriage and socio-economic status for women as compared with men (consistent with the idea that many benefits of marriage come from this association) and a greater effect of absence or loss of marriage on men compared with women (consistent with the idea that men are more dependent than women on the social support and social control elements of marriage). This can be seen as evidence for both protection and selection effects - marriage may bring socio-economic advantage and not getting married, or experiencing marriage termination, may be associated with characteristics that make attainment of socio-economic advantage less likely.

Effects of parity were also significant. We have previously reported results of analyses between women's reproductive histories and mortality (from age 50) and long-term illness in 1991, using the same data set but a different design and sample and taking account of factors such as length of birth intervals and timing of first and last births but not detailed marital history [39]. Overall, results from these two analyses are similar except that in our earlier work we found greater disadvantages associated with nulliparity (here positively associated with mortality, but not with long-term illness in 1991 and 2001). Results of another study, again using a sample drawn from this database but restricted to ever-married women, also showed raised mortality among nulliparous women; but no adverse effects of high parity, however this may have been because the investigators grouped all those with three or more children together [50]. The associations found probably reflect the influence of a range of factors, including selective influences (the childless and mothers of one child only may include women with health problems precluding successful first or second pregnancy and delivery); protective factors (social support from children); long-term sequelae of physiological challenges associated with high parity, and unmeasured characteristics of low and high parity women which may be associated with health and health related behaviors.

We did not explore interactions with age in any detail in this study but the fact that there were few significant associations between marital experience and long-standing illness in 2001 (when sample members were aged 70-89) but more indication of association in 1991 could be interpreted as a tendency towards convergence in differentials with older age. However, the smaller size of the 2001 sample and the fact that they represent a more selected group of survivors is also relevant, as are effects of earlier differential mortality more generally. We lacked information on mortality prior to 1971 (when those included in this study were aged 40-59), but mortality differences 1971-91 were explored in preliminary analysis and showed clear marital status differences. We investigated the influence of selection more formally by fitting a Heckman probit model [51] to presence of long-term illness in 2001 in which we included the same terms as in the logistic model reported in Table 4 and also a selection equation including marital status and socio-economic variables in 1971. This model takes account of the fact that those observed in 2001 represent only a selected sub set of the original 1971 population. Results showed that men who were never-married, widowed or divorced in 1971 were significantly less likely to be present in the 2001 sample; older age also reduced chances of inclusion in the 2001 analysis whereas owner occupation in 1971, access to a car and having an educational qualification was positively associated with survival to 2001. However, even allowing for this, coefficients for the 1971-91 marital history categories and other co-variates were very similar to those from models not including a selection term. Equivalent analysis for women showed that those who were divorced in 1971 (but not those then never-married or widowed) had significantly lower chances of survival to 2001 and that the effects of 1971 age and socio-economic status on inclusion in the 2001 sample were in the same direction as for men. Taking account of this selection had only slight effects on estimated covariates, although these were sufficient to make the reduced risk of 2001 long-term illness among women who had married for the first time since 1971 statistically significant in the Heckman probit model including a selection equation.

Strengths of this study include large sample size, availability of socio-economic indicators over several time points and in analyses for women, consideration of parity, inclusion of those in institutions (apart from the tiny proportion already resident in an institution before age 60) and low rates of non-response and loss to follow-up. However we were unable to precisely time marriages and divorces and lack information on childhood or early adult circumstances (other than an educational qualification indicator) which are likely to be associated both with marital trajectories and with later health. We also lacked data on health related behaviours and although we used several indicators of socio-economic status these were in some cases fairly crude. The measure of education available, for example, did not allow us to make any distinctions among the majority who only had lower level qualifications. It is therefore possible that some of the reported associations between marital history and status and health outcomes reflect residual confounding by socio-economic status. This may be particularly true for women as in the cohorts we consider the entwinement of female family trajectories with other aspects of the life course, including labour market participation and acquisition of wealth, makes disentangling their implications for health very difficult. As already discussed there are also limitations to the health information available. We had no information on health status in 1971 or 1981 and the data available for 1991 and 2001 were drawn from a single item question. A further limitation of this study, which is relevant more generally, is that despite the large size of the study sample, numbers in subgroups of interest were in some cases very small and power to detect differences between them consequently limited.

The cohorts we studied were born between 1912 and 1931. The earliest born within this range may have had marriage and fertility plans disrupted by war; the latest born are members of the 'marriage boom' generations with high rates of nuptiality, rising but still low rates of divorce and, on average, later widowhood than preceding generations. Cohorts born more recently have shown further changes in partnership behavior with later marriage, and more fluid partnership arrangements. Analyses of the implications of marital or partnership histories for later life health may be both more feasible and more relevant for these later born cohorts. Comparative studies of populations, such as those of the US and some Nordic countries, which already include higher proportions of older people with disrupted marital histories (because of the earlier adoption of more fluid and disrupted partnership arrangements), would also be useful. Such research is important because although changes in partnership patterns would suggest that the difference between married and unmarried is becoming less distinct, some large studies suggest that marital status differentials in mortality may be increasing [6,52] and we need to discover to what extent the changing marital history composition of marital status groups may account for this.

## Conclusion

Considering marital history, rather than current marital status alone, revealed higher health and mortality risks for women remarried after divorce, but not those remarried after widowhood, and also differences between men, (and women), who had remarried before age 59 and those remarried more recently. However, these groups were relatively small and in other respects additional insights gained from looking at marital history in this analysis were slight. However, for cohorts born more recently with more heterogeneous partnership patterns, this may be become more important. Effects were considerably modified by socio-economic status, particularly for women, lending support to studies which have suggested that for women health benefits of marriage may partly reflect socio-economic effects of marital status. For women, consideration of parity also influenced results suggesting that health implications of marital and fertility histories should be considered jointly.

## Competing interests

The authors declare that they have no competing interests.

## Authors' contributions

EG conceived of the study, participated in the design and statistical analysis and drafted the paper. CT undertook data preparation and participated in the design, statistical analysis and revisions of the draft. Both authors read and approved the final manuscript.

## Pre-publication history

The pre-publication history for this paper can be accessed here:

http://www.biomedcentral.com/1471-2458/10/554/prepub
